# Machine learning in developing a predictive model for chronic hydrocephalus following aneurysmal subarachnoid hemorrhage

**DOI:** 10.3389/fneur.2025.1651694

**Published:** 2026-01-12

**Authors:** Rao Dai, Enxi Xu, Lixiang Zhang, Zehui Wang, Bowen Liu, Siyuan Lu, Xiuhong Shan, Eryi Sun

**Affiliations:** 1 Department of Radiology, Affiliated People’s Hospital of Jiangsu University, Zhenjiang, Jiangsu, China; 2 Department of Neurosurgery, Affiliated People’s Hospital of Jiangsu University, Jiangsu, China; 3 Soochow Key Laboratory of Prevention and Treatment of Child Brain Injury, Children’s Hospital of Soochow University, Suzhou, China; 4 Jiangsu University of Science and Technology, Zhenjiang, Jiangsu, China

**Keywords:** clinical-radiological nomogram, chronic hydrocephalus, aneurysmal subarachnoid hemorrhage, 3D-Unet, machine learning

## Abstract

**Objective:**

Using machine learning (ML) algorithms integrated with deep learning and radiomics technologies, we developed a nomogram model through an in-depth analysis and mining of clinical data and imaging features from patients with aneurysmal subarachnoid hemorrhage (aSAH). This model was aimed to predict the risk of developing chronic hydrocephalus in aSAH patients.

**Methods:**

This study enrolled 410 patients diagnosed with subarachnoid hemorrhage (SAH) in the Neurosurgery Department of the Affiliated People’s Hospital of Jiangsu University between January 2020 and December 2023. Clinical and imaging characteristic data were collected from these patients. Using radiomic methods, we extracted features from the white matter surrounding the anterior horns of both lateral ventricles, ultimately selecting seven radiomic features to calculate the radiomics score. An automatic segmentation model based on the 3D-Unet architecture was specifically developed to measure hematoma volume. Initially, univariate analysis was conducted on all features, and the least absolute shrinkage and selection operator (LASSO) regression model was applied for feature selection using 10-fold cross-validation to optimize the penalty parameter. Key risk factors were identified, and various ML algorithms were used to construct and validate a predictive model, leading to the development of a clinical-radiological nomogram. To evaluate the model’s discriminative ability, we performed receiver operating characteristic (ROC) curve analysis and calculated the area under the curve (AUC). Additionally, the consistency between model predictions and actual outcomes was assessed using calibration curves. Further evaluation included plotting precision-recall (P-R) curves, decision curve analysis (DCA), and clinical impact curves (CIC) to demonstrate the net benefit of the model at various thresholds in the training and test sets, validating its clinical utility.

**Results:**

A total of 180 patients were included, and a 3D-Unet automatic segmentation model was developed to accurately identify and quantify SAH volume. In the test set, the model achieved a Dice similarity coefficient (DSC) of 0.85 ± 0.04, an intersection over union (IoU) of 0.74 ± 0.06, a Hausdorff distance (HD) of 20.4 ± 12.3, and an average symmetric surface distance (ASSD) of 0.31 ± 0.23, demonstrating excellent performance in identifying SAHs. After screening features such as hematoma volume and radiomic score through univariate logistic regression (LR), 21 potential risk factors were identified. LASSO regression further refined these to nine key risk factors. Combining the results from both analyses, 6 independent predictive factors were determined: cerebrospinal fluid lactic acid level, sodium (Na), corpus callosum angle, interval to blood clearance, periventricular white matter changes, and hematoma volume. Among 8 ML models, the LR model showed the best performance, with AUC values of 0.884 [95% confidence interval (CI): 0.826–0.942] in the training cohort and 0.860 (95% CI: 0.758–0.962) in the test cohort. The calibration curve of the LR model showed a high agreement between predicted probabilities and observed outcomes. Additionally, DCA and CIC analyses demonstrated significant net benefits across different risk thresholds, confirming high consistency between predictions and actual outcomes.

**Conclusion:**

The developed 3D-Unet automatic segmentation model accurately identified hematomas and calculated their volume, addressing the challenge of quantitatively assessing SAH volume in clinical practice. Hematoma volume, a key risk factor, was integrated with clinical and radiological features from computed tomography (CT) scans using ML methods to construct a clinical-radiological nomogram. This nomogram effectively predicted the development of chronic hydrocephalus in patients with aSAH.

## 1 Introduction

Aneurysmal subarachnoid hemorrhage (aSAH) is a common cause of hemorrhagic stroke (1), with a pre-hospital mortality rate ranging from 22 to 26% (2). Most survivors experience significant functional morbidity and neurocognitive deficits (2). Hydrocephalus is a frequent complication of aSAH and can result in worse neurocognitive outcomes and higher mortality rates (3). Chronic hydrocephalus develops in 7–48% of aSAH patients after 14 days (4). Chronic hydrocephalus can lead to cognitive impairment, gait abnormalities, incontinence, epilepsy, and visual disturbances (5). If not diagnosed and treated promptly, chronic hydrocephalus may result in irreversible neurological dysfunction (6, 7). Therefore, early prediction of chronic hydrocephalus in aSAH patients is very important.

In a previous study (8), we analyzed the correlations between general patient characteristics, imaging indicators, laboratory test results, surgical factors, and acute hydrocephalus. We identified three factors periventricular white matter changes, external lumbar drainage, and the modified Fisher grade as being closely related to the occurrence of chronic hydrocephalus after aSAH. Building upon these findings, the present study further investigated these aspects. Radiomics enables the extraction of numerous quantitative features from imaging data and analyzes the correlation between these features and disease diagnosis or prognosis (9, 10). For example, Houman Sotoudeh et al. used radiomic features extracted from the lateral ventricles on T2-weighted images to predict the treatment responsiveness of hydrocephalus (11). Other studies have demonstrated that cerebral hemorrhage and hydrocephalus can damage the periventricular white matter (12, 13). Therefore, we attempted to extract radiomic features from the periventricular regions of both lateral ventricles and evaluate their correlation with the formation of chronic hydrocephalus. The modified Fisher grade is a method used to assess the severity of SAH on non-contrast computed tomography images (14). It is susceptible to observer variability and lacks the ability to accurately quantify hemorrhage volume (15). Recent studies have investigated the relationships between subarachnoid hematoma, intraventricular hemorrhage, clinical complications, and patient outcomes using deep learning-based automatic segmentation methods (16, 17). However, few studies have explored the correlation between total hemorrhage volume at admission and the development of chronic hydrocephalus in patients with aSAH.

Machine learning (ML) has been widely applied in medicine, particularly for diagnosis, prognosis, and predicting treatment outcomes. In recent years, research teams have adopted ML algorithms to identify the formation of Hydrocephalus (18–21). Convolutional neural networks (CNNs) was also used in this study. The 3D CNN architecture, commonly used for medical image segmentation, is characterized by a U-shaped structure with a contraction path for capturing contextual information and an expansion path for precise localization. This architecture effectively combines low-level and high-level features, preserving finer image details and achieving robust learning with limited data (22). Emerging studies have demonstrated the utility of 3D CNNs in classifying cerebral hemorrhages (23, 24).

In this study, we employed a ML model based on clinical and neuroimaging data, integrating radiomics methods and a 3D-Unet deep learning framework for automatic segmentation. Specifically, we investigated periventricular white matter changes in patients with chronic hydrocephalus following aSAH and quantitatively calculated hemorrhage volume at admission. Our aim was to develop a predictive model for the early identification of chronic hydrocephalus in aSAH patients, thereby enabling clinicians to intervene early for high-risk individuals and mitigate the adverse effects of chronic hydrocephalus.

## 2 Methods

### 2.1 Study design and patients

Our study enrolled 410 patients with aSAH admitted to the Neurosurgery Department of the Affiliated People’s Hospital of Jiangsu University between January 2020 and December 2023. Data were retrospectively collected using a clinical research data platform. Patients were excluded if they did not have aneurysms, had inadequate imaging quality, were hospitalized for fewer than 5 days, refused participation, died within 30 days of treatment, had other brain diseases (e.g., vascular malformations, brain tumors), or had a history of hydrocephalus or shunt surgery. *Diagnostic Criteria for Chronic Hydrocephalus*: 1. *Time criterion*: Defined as 14 days or more after subarachnoid hemorrhage (SAH). 2. *Imaging criteria*: Meeting at least one of the following: Evans index >0.30, callosal angle <90°, or unilateral/bilateral temporal horn width >2 mm. 3. *Exclusion criteria*: Congenital hydrocephalus, cerebral atrophy, brain tumors, or other causes of ventricular enlargement were excluded. Hospitalization duration <5 days (82 cases).

**Figure 1:**
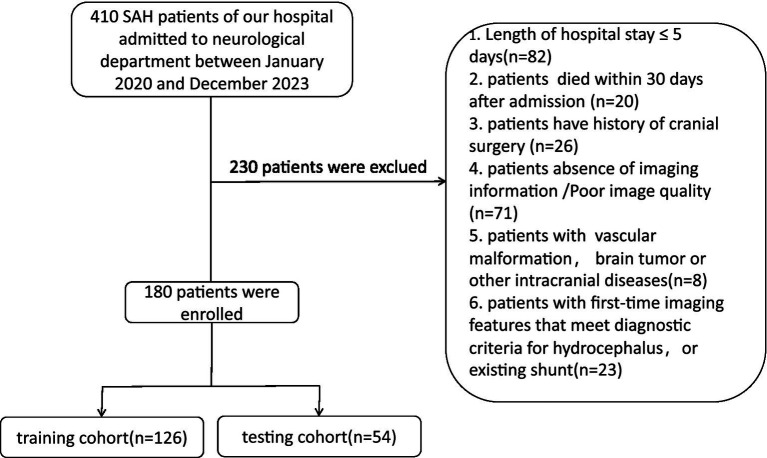
Recruitment pathway for eligible patients in this study.

Death within 30 days (20 cases); History of cranial surgery (26 cases); Missing or poor-quality imaging data (71 cases); Presence of other intracranial diseases (8 cases); History of pre-existing hydrocephalus or shunt surgery (23 cases). After rigorous screening and data organization, medical records from 180 patients were included in the statistical analysis and randomly assigned to a training cohort and a validation cohort in a 7:3 ratio (Figure 1). This retrospective study was approved by the Medical Ethics Committee of the Affiliated People’s Hospital of Jiangsu University (Approval Number: K-202400164 W) and conducted in adherence to the principles of the Helsinki Declaration. As all patient data were anonymized and de-identified prior to analysis, informed consent was not required.

### 2.2 Clinical and surgical data

This study meticulously extracted demographic and clinical data from the medical record system. In addition to recording patients’ basic information, we assessed comorbidities, including hypertension [as described in our previous study (8)], diabetes, heart disease, stroke, and prior history of surgery. Key clinical indicators, such as the Glasgow Coma Scale (GCS) score, Hunt and Hess grade, World Federation of Neurosurgical Societies (WFNS) grade, and modified Fisher grade at admission, were also documented. The diagnosis of delayed cerebral ischemia (DCI) was based on established field standards, incorporating clinical symptoms (new-onset neurological deficits, impaired consciousness) combined with imaging evidence of ischemia (new infarct foci indicated by CT). We organized examination data to include the location of aneurysms, surgical procedures, and the presence of craniectomy and ventricular hemorrhage for each patient. The blood clearance estimated are as follows: The timeline starts from hospital admission (or symptom onset) and continues until follow-up CT scans show no significant high-density blood signals in the subarachnoid space and ventricular system (as independently assessed by two radiologists, with disagreements resolved by a third evaluator). The first day on which “complete absorption” is observed is recorded as the “clearance interval.”

Postoperative data included details about lateral ventricular drainage, ventriculoperitoneal (V-P) shunt, and lumbar puncture drainage. Complications were also recorded, such as acute or subacute hydrocephalus, cerebral hernia, and delayed cerebral ischemia and the time required for the complete clearance of intracranial hematomas. All data were rigorously reviewed by clinicians to ensure accuracy and consistency. Within the first week of admission, cerebrospinal fluid (CSF) tests were performed multiple times to measure chloride, glucose, cell count, and protein levels. Measurement was taken within 7 days after admission, using the results from the first lumbar puncture (LP) or external ventricular drainage (EVD) specimen. The 7-day window was chosen because it corresponds to the critical phase of arachnoid inflammation and CSF circulation abnormalities triggered by blood breakdown products. For serological indicators, the reference ranges were: white blood cell count 3.5–9.5 × 10^9^/L and C-reactive protein (CRP) 0–5 mg/L. Electrolyte levels were also monitored, with reference ranges as follows: calcium (Ca) 2.08–2.66 mmol/L, potassium (K) 3.5–5.1 mmol/L, sodium (Na) 135–145 mmol/L, and chloride (Cl) 95–106 mmol/L. Measurement was taken within 24 h after admission, using the first test result as the analytical value. If multiple tests were performed within 24 h, the earliest baseline data were selected. Radiological features were obtained by manually measuring the initial CT scan images upon admission, as described in our previous study (8).

### 2.3 Calculation of radiomics score (radscore)

**Figure 2:**
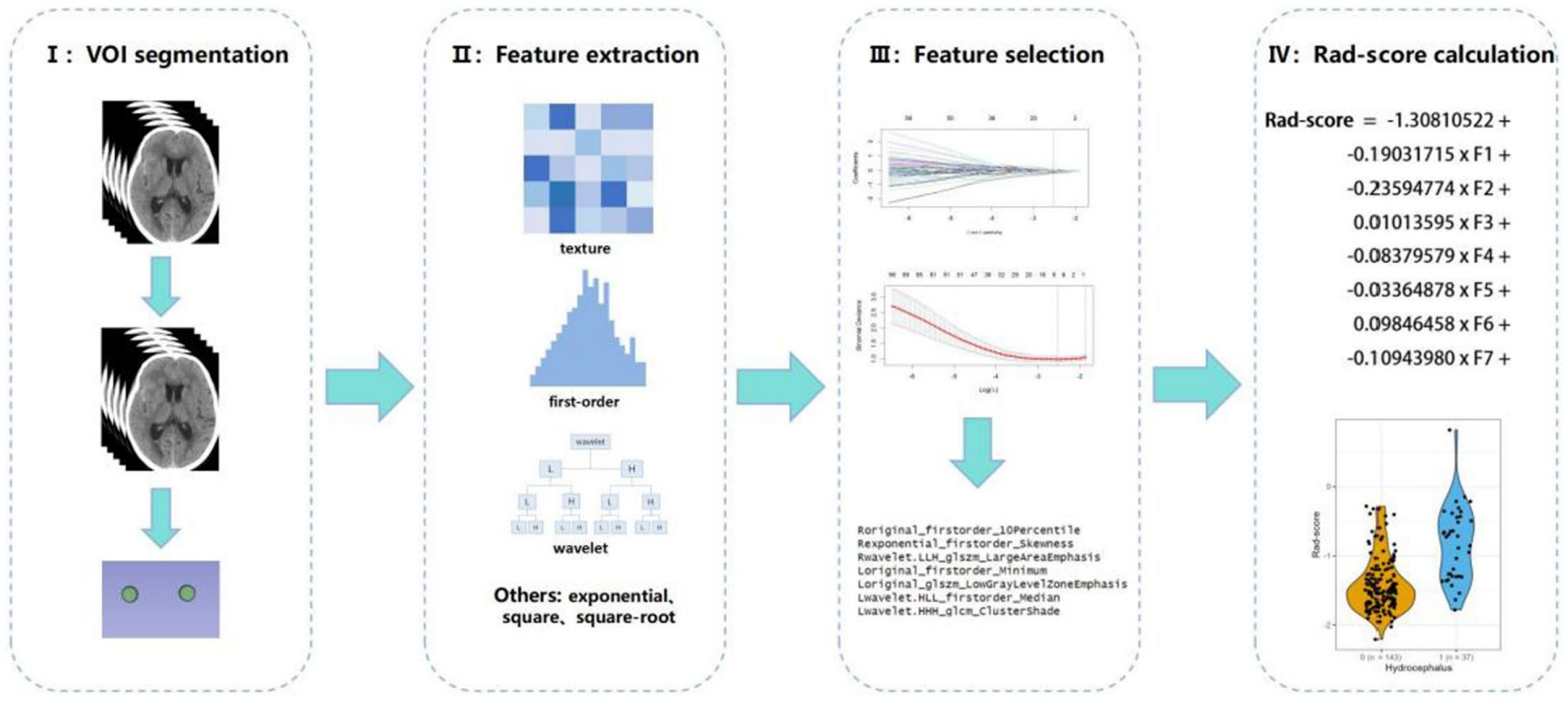
Radiomics workflow diagram.

Due to the relatively concealed formation process of hydrocephalus, it is difficult to detect it through a single CT scan. As noted in our previous study, white matter surrounding the ventricles changed after 7 days post-SAH (8). For the calculation of radscore, CT scans from the 7th day of admission were selected. The region of interest (ROI) was defined as the periventricular white matter within a 1 cm diameter around the anterior horns of the lateral ventricles. CT images in DICOM format were imported into 3D Slicer (version 5.2.2), and the delineation of ROI was performed by experienced resident physicians and validated by a senior radiologist with over 20 years of experience in diagnosing brain diseases. Radiomic features were extracted from the periventricular white matter around the anterior horns of both lateral ventricles using Pyradiomics (25), yielding 1,395 features per ROI after excluding morphological features. The features extracted from both the left and right sides were collectively compared between the two patient groups using the Mann–Whitney U test, and those with *p* ≥ 0.05 were excluded. Least absolute shrinkage and selection operator (LASSO) regression, with 10-fold cross-validation, identified the optimal regularization parameter (*λ*), an intercept (*α*), and the corresponding coefficients (*β*) for each feature. Seven features and their corresponding coefficients were retained for calculating the final radiomics score (Figure 2).

### 2.4 3D-Unet automatic segmentation model

**Figure 3:**
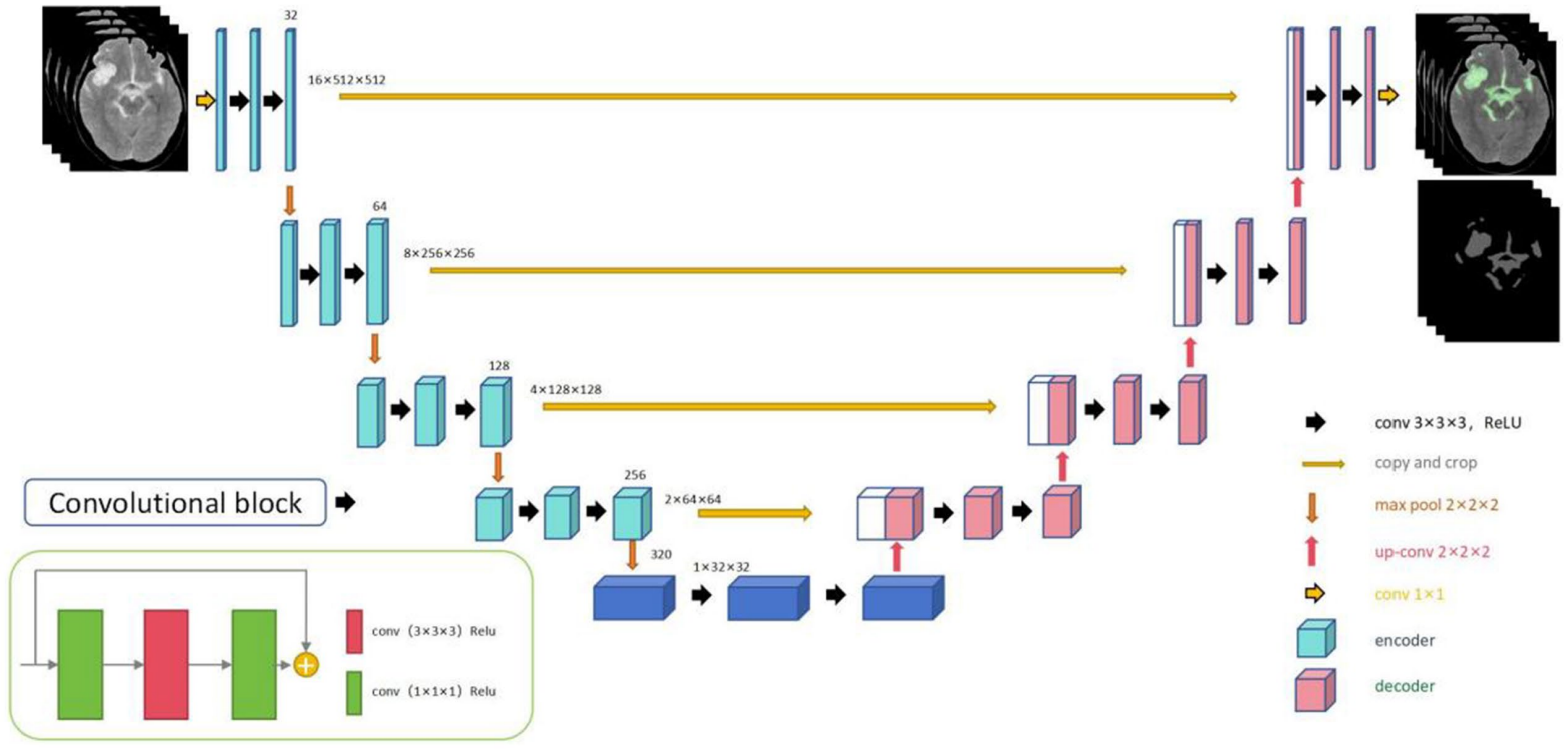
Framework diagram of the 3D-Unet automatic segmentation model developed in this study.

The 3D-Unet automatic segmentation model (Figure 3) was trained using initial CT scan images obtained at admission. The images were standardized and resampled to a uniform spatial resolution of 0.5 × 0.5 × 5 mm. After removing the skull, hemorrhagic lesions were annotated slice by slice. The dataset was split into training, validation, and testing sets in a 7:1:2 ratio. Data diversity was enhanced by horizontal flipping of the training dataset. Detailed parameters of the radiomics and segmentation models are provided in Supplementary material.

### 2.5 Selection of risk factors

Univariate logistic regression (LR) was employed to analyze clinical and radiological parameters. Significant attributes (*p* < 0.05) were further analyzed using LASSO regression with 10-fold cross-validation to refine the selection. Multivariate LR identified significant risk factors for chronic hydrocephalus after aSAH (13). These factors were integrated into predictive models using both LR and ML algorithms, including extreme gradient boosting (XGB), random forest (RF), support vector machine (SVM), decision tree (DT), and k-nearest neighbor (KNN). A user-friendly nomogram was developed for healthcare professionals (26, 27).

### 2.6 Statistical analysis

Statistical analyses were conducted using SPSS 22.0 and R software (version 4.1) (8, 13). Continuous variables were presented as median [interquartile ranges (IQR)] or mean ± standard deviation (SD), depending on the results of the Shapiro–Wilk normality test. Categorical data were expressed as proportions. Chi-square tests, Fisher’s exact tests, and Mann–Whitney U tests were used to assess differences among cohorts. Univariate LR analysis was conducted to identify potential risk factors associated with the study outcomes, with variables showing a *p* < 0.05 included in a stepwise multivariable LR model. The nomogram’s predictive performance was evaluated by calculating the area under the receiver operating characteristic (ROC) curve (AUC), comparing predicted probabilities to actual outcomes. Calibration curves were used to assess model fidelity, and goodness-of-fit testing was performed, with calibration performance measured using Boullier scores. Clinical utility of the refined model was further evaluated using decision curve analysis (DCA) across different probability thresholds for both groups. ML algorithms, renowned for their superior predictive performance, have demonstrated superior outcomes compared to traditional regression methods for large datasets (28). To enhance predictive accuracy, six ML algorithms were applied: XGB, LR, RF, SVM, deep regression (DR), and KNN. For model training, 70% of the dataset was randomly selected, while the remaining 30% was reserved for testing. To mitigate overfitting, appropriate adjustments were made during training, and optimal hyperparameters were determined using five-fold cross-validation. The optimized model was implemented in R to predict the risk of chronic hydrocephalus following aSAH. Statistical significance was defined as a *p* < 0.05.

## 3 Results

### 3.1 Patient characteristics

A retrospective analysis was conducted using data from 410 patients enrolled at our institution, of whom 180 were included in this study. The cohort was randomly divided into training and testing groups in a 7:3 ratio. No significant differences were found across all variables between the training and testing groups (Table 1) (*p* > 0.05). The overall incidence of chronic hydrocephalus was 22.2% (40/180), with an incidence of 24.8% (31/125) in the training group and 16.3% (9/55) in the testing group.

**Table 1:** The baseline characteristics of the enrolled patients in the training and test cohorts.

Characteristics	(ALL) *N* = 180	Test *N* = 55	Train *N* = 125	p.overall
Group	0.22 (0.42)	0.16 (0.37)	0.25 (0.43)	0.187
Gender: n(%)				0.750
Female	116 (64.44%)	34 (61.82%)	82 (65.60%)	
Male	64 (35.56%)	21 (38.18%)	43 (34.40%)	
Age (years) media [Q1; Q3]	58.50 [52.75; 67.25]	61.00 [52.50; 68.50]	58.00 [53.00; 67.00]	0.510
Past medical history				
Ischemic stroke, n(%)				1.000
Negative	176 (97.78%)	54 (98.18%)	122 (97.60%)	
Positive	4 (2.22%)	1 (1.82%)	3 (2.40%)	
Hypertension: n(%)				0.767
Negative	93 (51.67%)	27 (49.09%)	66 (52.80%)	
Positive	87 (48.33%)	28 (50.91%)	59 (47.20%)	
Heart disease: n(%)				1.000
Negative	173 (96.11%)	53 (96.36%)	120 (96.00%)	
Positive	7 (3.89%)	2 (3.64%)	5 (4.00%)	
Diabetes: n(%)				0.191
Negative	168 (93.33%)	49 (89.09%)	119 (95.20%)	
Positive	12 (6.67%)	6 (10.91%)	6 (4.80%)	
Surgical method: n(%)				0.857
Coiling	98 (54.44%)	31 (56.36%)	67 (53.60%)	
Clipping	82 (45.56%)	24 (43.64%)	58 (46.40%)	
Craniectomy: n(%)				0.874
Negative	151 (83.89%)	47 (85.45%)	104 (83.20%)	
Positive	29 (16.11%)	8 (14.55%)	21 (16.80%)	
Location of aneurysm: n(%)				1.000
Anteria	18 (10.00%)	5 (9.09%)	13 (10.40%)	
Posterior	162 (90.00%)	50 (90.91%)	112 (89.60%)	
External lumbar drainage: n(%)				0.862
Negative	147 (81.67%)	44 (80.00%)	103 (82.40%)	
Positive	33 (18.33%)	11 (20.00%)	22 (17.60%)	
Hunt and Hess grade, media [Q1; Q3]	2.00 [1.00; 3.00]	2.00 [1.00; 2.50]	2.00 [1.00; 3.00]	0.571
GCS score, media [Q1; Q3]	15.00 [12.00; 15.00]	15.00 [12.00; 15.00]	15.00 [11.00; 15.00]	0.496
WFNS grade, media [Q1; Q3]	1.00 [1.00; 4.00]	1.00 [1.00; 4.00]	1.00 [1.00; 4.00]	0.442
Acute hydrocephalus: n(%)				0.094
Negative	169 (93.89%)	49 (89.09%)	120 (96.00%)	
Positive	11 (6.11%)	6 (10.91%)	5 (4.00%)	
Subacute hydrocephalus: n(%)				0.497
Negative	170 (94.44%)	51 (92.73%)	119 (95.20%)	
Positive	10 (5.56%)	4 (7.27%)	6 (4.80%)	
V-P shunt, n(%)				1.000
Negative	177 (98.33%)	54 (98.18%)	123 (98.40%)	
Positive	3 (1.67%)	1 (1.82%)	2 (1.60%)	
EVD placement, n(%)				0.819
Negative	157 (87.22%)	47 (85.45%)	110 (88.00%)	
Positive	23 (12.78%)	8 (14.55%)	15 (12.00%)	
Intracranial infection: n(%)				1.000
Negative	166 (99.40%)	51 (100.00%)	115 (99.14%)	
Positive	1 (0.60%)	0 (0.00%)	1 (0.86%)	
Callosal angle, media [Q1; Q3]	111.30 [104.27; 117.60]	110.50 [103.50; 117.35]	112.00 [104.90; 117.60]	0.652
Intraventricular bleeding: n(%)				0.891
Negative	105 (58.33%)	33 (60.00%)	72 (57.60%)	
Positive	75 (41.67%)	22 (40.00%)	53 (42.40%)	
Modified fisher grade, n(%)				0.242
1	55 (30.56%)	12 (21.82%)	43 (34.40%)	
2	69 (38.33%)	24 (43.64%)	45 (36.00%)	
3	49 (27.22%)	18 (32.73%)	31 (24.80%)	
4	7 (3.89%)	1 (1.82%)	6 (4.80%)	
Interval to blood clearance (days), media [Q1; Q3]	16.00 [11.00; 26.25]	17.00 [13.00; 29.00]	16.00 [11.00; 25.00]	0.206
Evans index, media [Q1; Q3]	0.26 [0.24; 0.29]	0.26 [0.24; 0.28]	0.26 [0.24; 0.29]	0.740
Third ventricle width (mm), media [Q1; Q3]	6.00 [5.00; 8.00]	6.00 [4.91; 8.00]	6.00 [5.00; 8.00]	0.976
Periventricular white matter changes, n(%)				0.805
Negative	127 (70.56%)	40 (72.73%)	87 (69.60%)	
Positive	53 (29.44%)	15 (27.27%)	38 (30.40%)	
Subdural hygroma, n(%)				0.848
Negative	131 (72.78%)	39 (70.91%)	92 (73.60%)	
Positive	49 (27.22%)	16 (29.09%)	33 (26.40%)	
Radscore, media [Q1; Q3]	−1.42 [−1.67; -1.04]	−1.52 [−1.71; -1.07]	−1.40 [−1.63; -1.04]	0.166
Hematoma of volume (mL), media [Q1; Q3]	26.82 [11.37; 58.94]	32.32 [13.52; 57.34]	25.35 [11.12; 59.58]	0.409
Cerebral hernia, n(%)				1.000
Negative	172 (96.09%)	53 (96.36%)	119 (95.97%)	
Positive	7 (3.91%)	2 (3.64%)	5 (4.03%)	
Delayed cerebral ischemia (DCI), n(%)				0.928
Negative	131 (73.18%)	41 (74.55%)	90 (72.58%)	
Positive	48 (26.82%)	14 (25.45%)	34 (27.42%)	
Lactic acid (mmol/L), media [Q1; Q3]	2.00 [1.50; 3.00]	1.90 [1.50; 3.25]	2.00 [1.50; 2.90]	0.575
Respiratory failure, n(%)				0.898
Negative	157 (87.71%)	49 (89.09%)	108 (87.10%)	
Positive	22 (12.29%)	6 (10.91%)	16 (12.90%)	
k (3.5–5.1 mmol/L), media [Q1; Q3]	3.76 [3.49; 4.09]	3.83 [3.50; 4.10]	3.73 [3.49; 4.08]	0.625
Na (135–145 mmol/L), media [Q1; Q3]	140.90 [137.88; 144.62]	140.60 [138.20; 143.85]	141.40 [137.80; 144.70]	0.574
Cl (95–106 mmol/L), media [Q1; Q3]	100.50 [97.40; 104.65]	100.30 [98.10; 103.30]	100.60 [97.10; 104.90]	0.657
Ca (2.08–2.66 mmol/L), media [Q1; Q3]	2.19 [2.10; 2.32]	2.20 [2.10; 2.30]	2.18 [2.10; 2.32]	0.765
CSF cell count (10^6^/L), media [Q1; Q3]	64.00 [12.75; 231.50]	64.00 [16.00; 217.50]	62.00 [13.00; 236.00]	0.947
CSF glucose (mmol/L), media [Q1; Q3]	4.00 [3.34; 4.02]	4.00 [3.55; 4.13]	4.00 [3.26; 4.00]	0.226
CSF chlorides (mmol/L), media [Q1; Q3]	125.50 [119.18; 128.00]	126.30 [120.80; 128.00]	124.90 [118.80; 128.00]	0.599
CSF protein(g/L), media [Q1; Q3]	0.47 [0.21; 1.54]	0.44 [0.21; 1.47]	0.50 [0.21; 1.54]	0.732

### 3.2 Radscore

The final seven selected radiomic features and their corresponding coefficients, were used to calculate the radiomic score (Supplementary Figure 1; Table 2).

**Table 2:** The seven retained radiomic features and their coefficients.

Features name	Coefficients
original_firstorder_10Percentile	−0.19031715
exponential_firstorder_Skewness	−0.23594774
wavelet. LLH_glszm_LargeAreaEmphasis	0.01013595
original_firstorder_Minimum	−0.08379579
original_glszm_LowGrayLevelZoneEmphasis	−0.03364878
wavelet. HLL_firstorder_Median	0.09846458
wavelet. HHH_glcm_ClusterShade	−0.10943980

**Figure 4:**
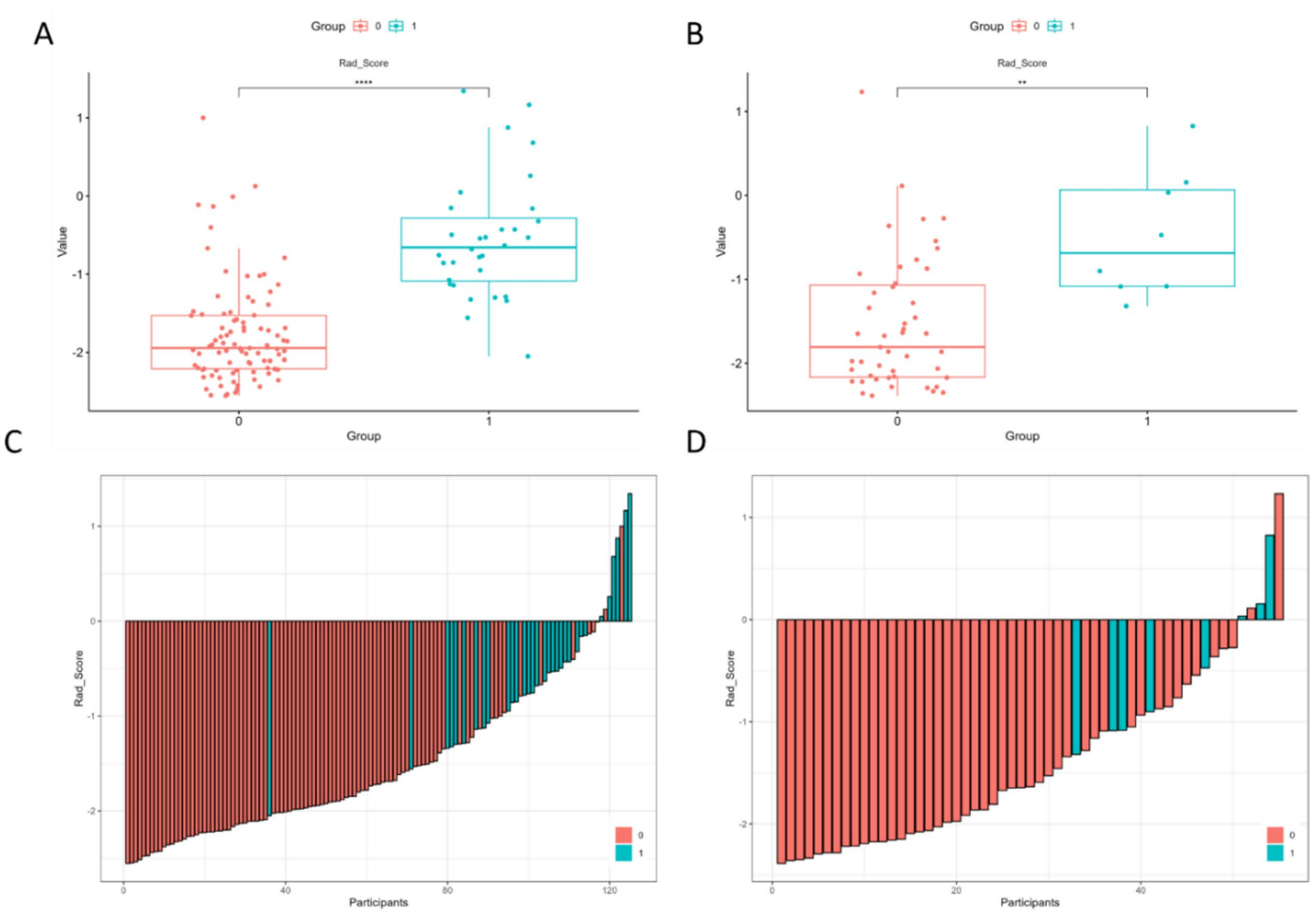
Distribution of radscores in both the training and test sets. **(A,B)** Box plot of radscores in both the training and the test sets, comparing patients with and without chronic hydrocephalus. **(C,D)** Waterfall plot showing the consistency of rad-scores across the two groups in both the training and test sets. (0 = Negative, 1 = positive).

Waterfall plots and box plots were used to show the distribution of the radscore in both the training and test cohorts (Figure 4).

These findings highlighted the consistent performance between the two cohorts and effectively demonstrated how the radiomic features differentiated between outcome groups.

### 3.3 3D-Unet automatic segmentation model

The training process and internal validation results of the 3D-Unet automatic segmentation model are shown in Supplementary Figure 2. Following training, 36 images were used as the test set to quantitatively evaluate the segmentation performance of the model using several evaluation metrics: Dice similarity coefficient (DSC), Intersection over Union (IoU), Hausdorff Distance (HD), and Average Symmetric Surface Distance (ASSD). The performance metrics for the model are presented in Supplementary Table 1.

### 3.4 Selection of risk factors and model construction

**Figure 5:**
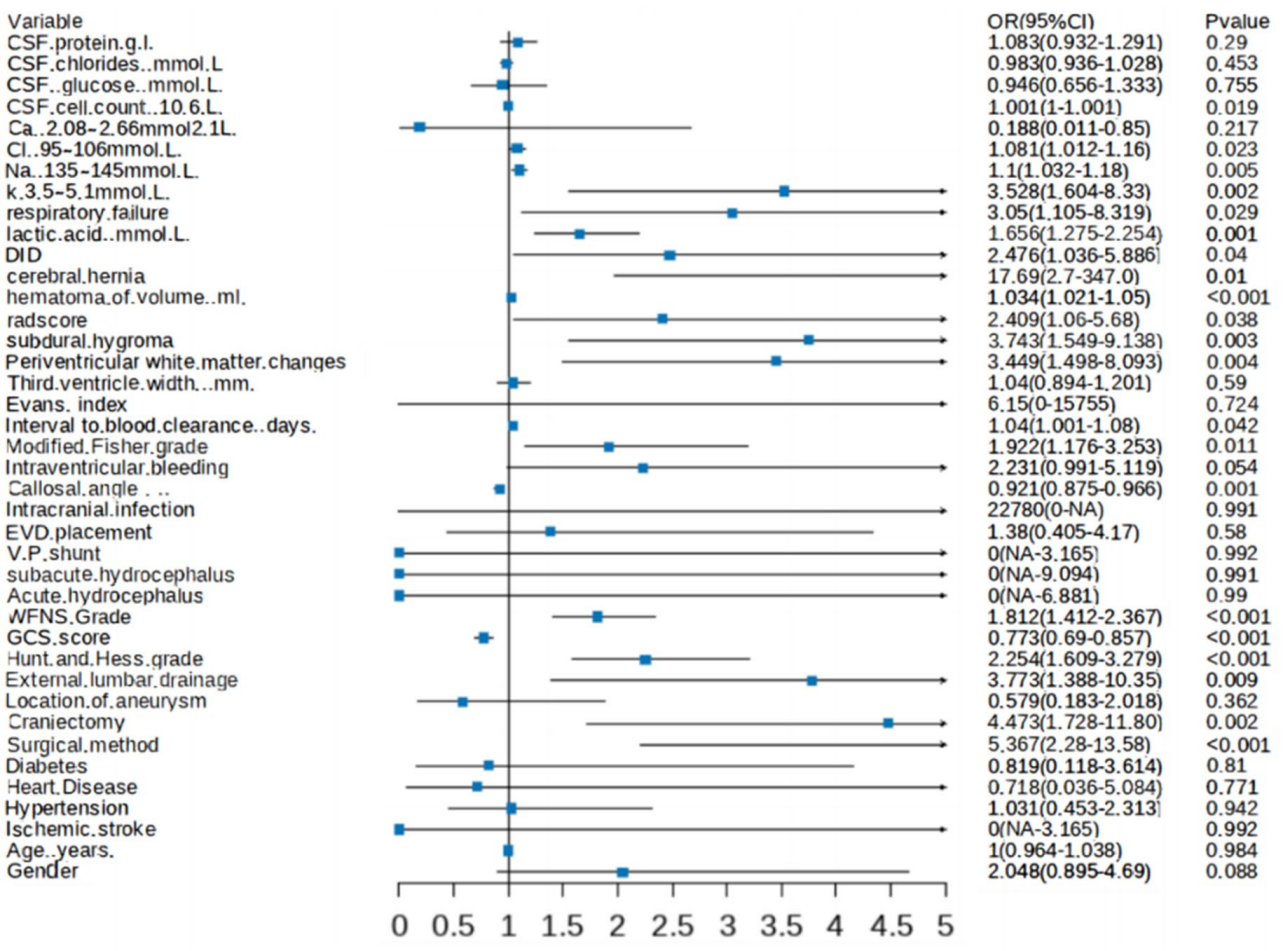
Forest plot showing characteristics identified through univariate logistic regression analysis.

A forest plot was generated to visually illustrate the various risk factors associated with chronic hydrocephalus after aSAH using univariate LR analysis (Figure 5).

**Figure 6:**
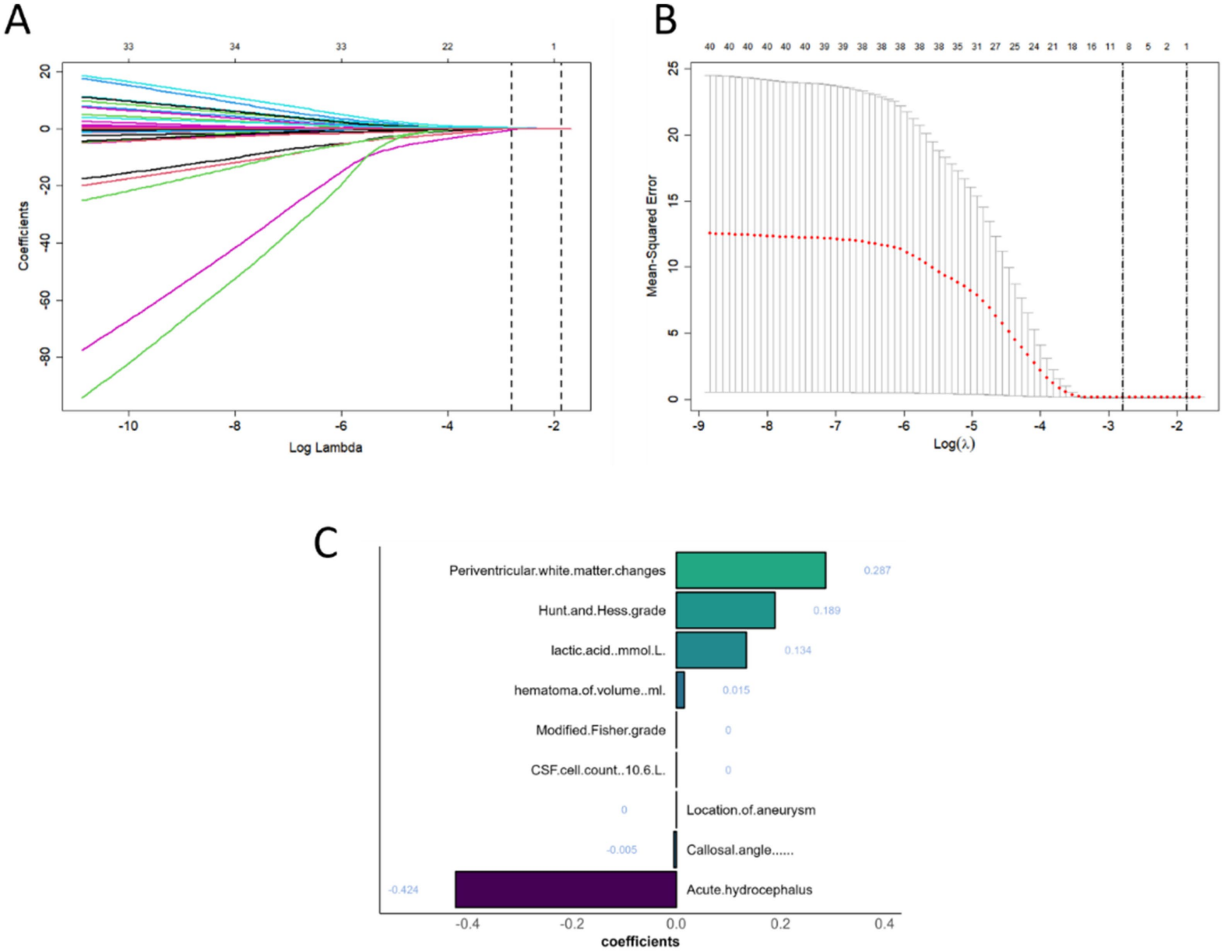
Patient characteristic selection using the LASSO for binary logistic regression model. **(A)** LASSO coefficient profiles for clinical and radiological features. **(B)** The optimal penalty coefficient lambda determined by LASSO through 10-fold cross-validation, with the corresponding mean squared error for the training cohort. **(C)** Characteristics selected by LASSO and retained in the model.

This analysis identified 21 risk factors with *p* < 0.05, and LASSO regression was used to determine nine prominent factors associated with chronic hydrocephalus after aSAH (Figure 6).

Ultimately, six key determinants were identified: cerebrospinal fluid lactic acid level, Na, corpus callosum angle, interval to blood clearance, periventricular white matter changes, and hematoma volume. These factors were further analyzed using a combination of ML algorithms, including LR, XGB, LGBM, RF, SVM, DT, and KNN. In the training set, KNN, RF, and XGB models exhibited the highest performance, achieving AUC values of 1.00, 1.00, and 0.96, respectively, indicating exceptional predictive capabilities. Other models, such as LR and SVM, also performed well.

However, in the test set, the performance of some models declined. KNN and RF, in particular, appeared to suffer from overfitting, making them unsuitable for further evaluation in subsequent model-building processes. The SVM and LR models demonstrated greater stability, with minimal variation in performance across different datasets.

The LGBM model showed poor performance in both the training and test sets, struggling to distinguish between samples.

**Figure 7:**
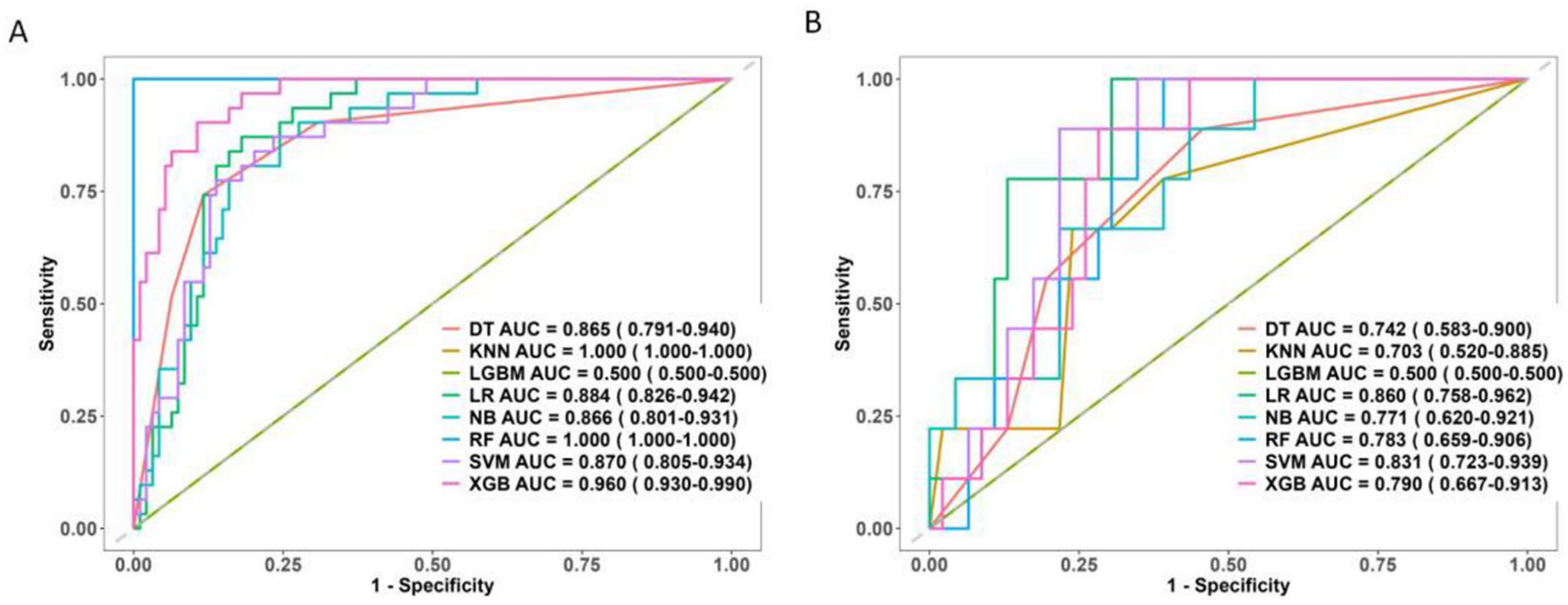
ROC curve of 8 machine learning models. **(A)** The training set. **(B)** The test set.

Therefore, when selecting a model for predicting chronic hydrocephalus, it is crucial to account for potential overfitting. While some models performed exceptionally well in the training set, LR, SVM, NB, and DT demonstrated more robust performance with better generalization. LR outperformed other models in the test set, making it the preferred choice for predictive tasks (Figure 7).

### 3.5 Model evaluation and clinical application

**Figure 8:**
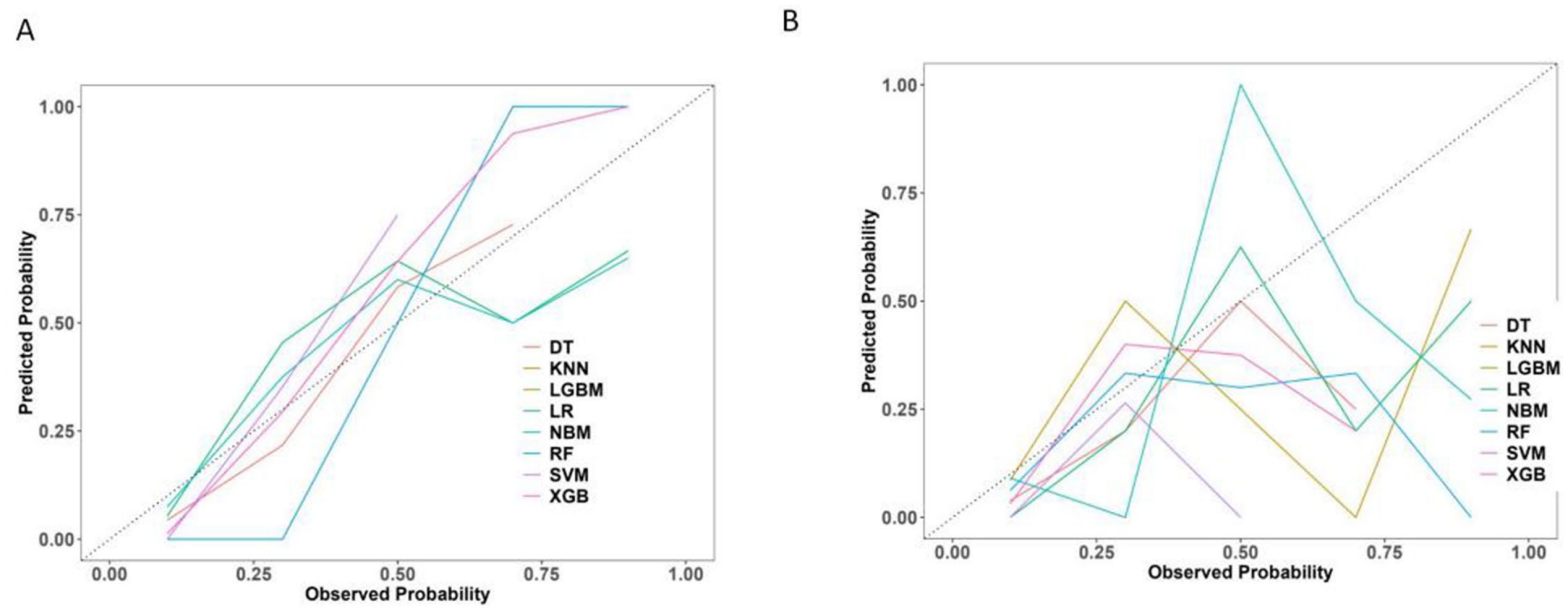
Calibration curve of 8 machine learning models. **(A)** The training set. **(B)** The test set.

The calibration curves revealed that both the LR and SVM models showed relatively stable performance across both the training and test datasets. The slight variation between their predicted and actual probabilities highlighted their strong generalization capabilities. In contrast, while the DT and XGB models performed well in the training dataset, their performance significantly deteriorated in the test dataset, suggesting a significant risk of overfitting (Figure 8).

**Figure 9:**
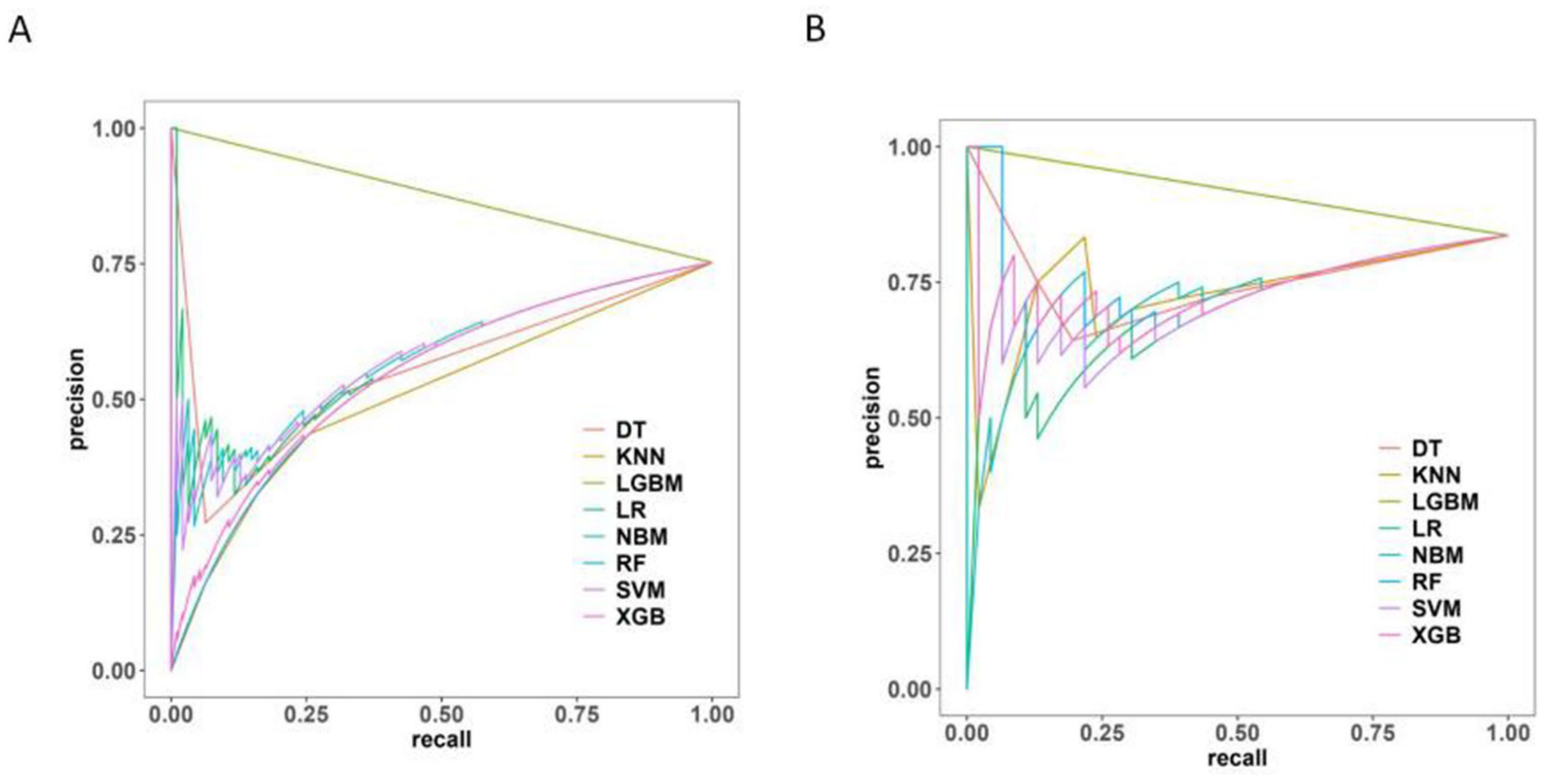
Precision-recall curve of 8 machine learning models. **(A)** The training set. **(B)** The test set.

The Precision-Recall (P-R) curve (Figure 9) demonstrated a balance between precision (positive predictive value) and recall (sensitivity), which might be particularly valuable in evaluating classifiers in imbalanced class situations.

**Figure 10:**
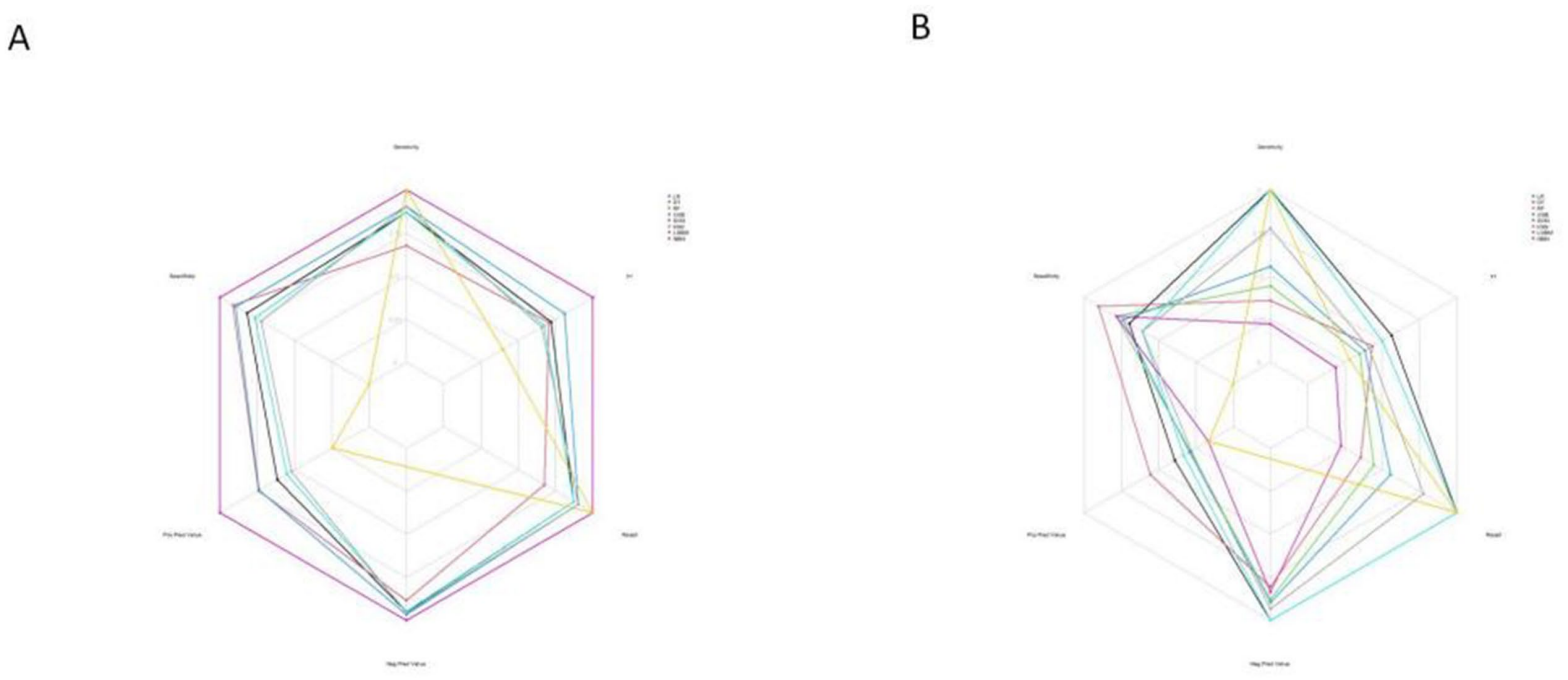
Radar chart of confusion matrix evaluation indexes for 8 machine learning models. **(A)** The training set. **(B)** The test set.

Furthermore, the calibration curves for the LR model in both the training and test sets showed optimal performance (Figure 10), indicating strong consistency between the models’ predictions and actual outcomes.

**Figure 11:**
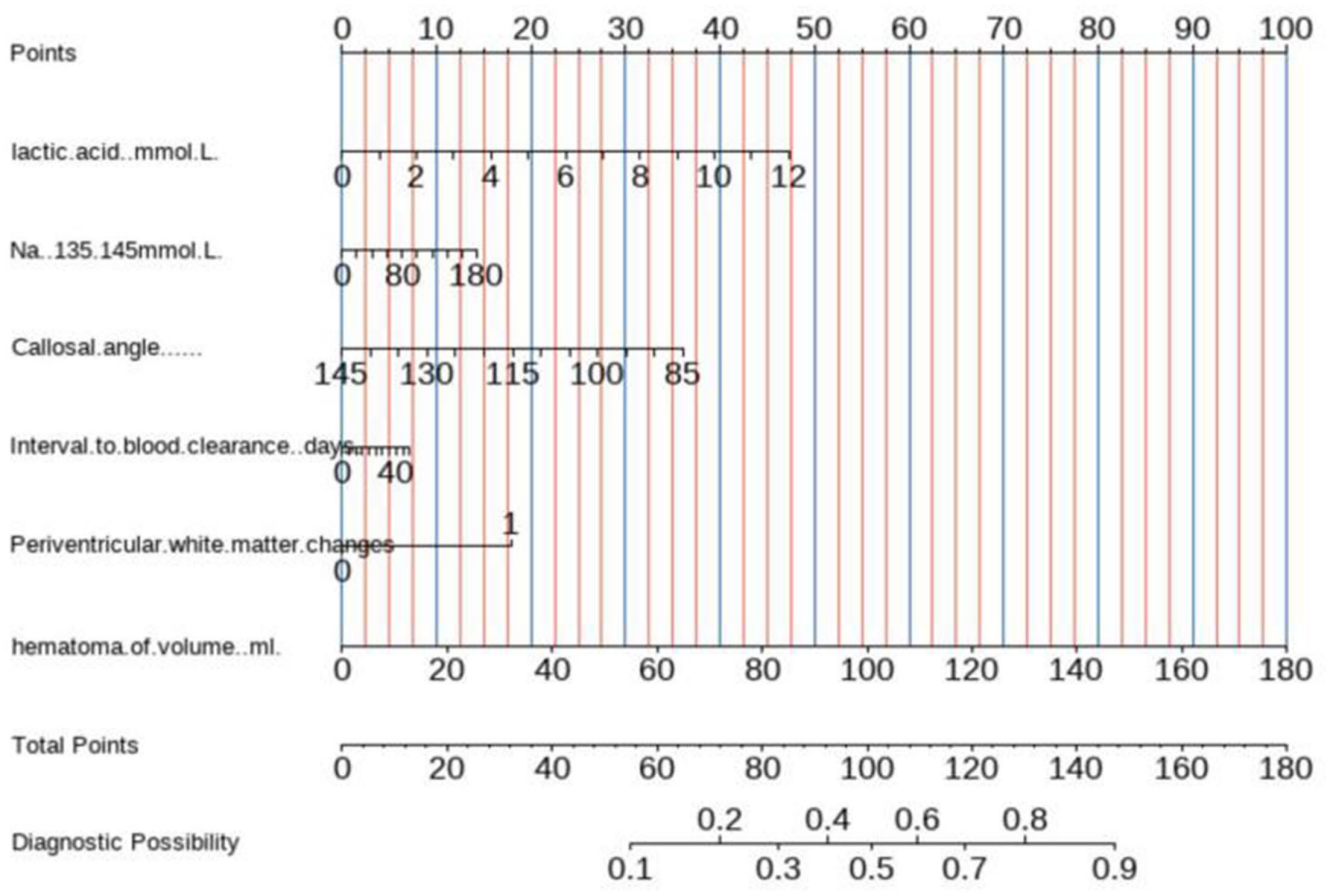
Nomogram constructed from characteristics jointly selected through multivariate logistic regression analysis.

Based on these analyses, we developed a nomogram to predict the likelihood of chronic hydrocephalus after aSAH (Figure 11).

**Figure 12:**
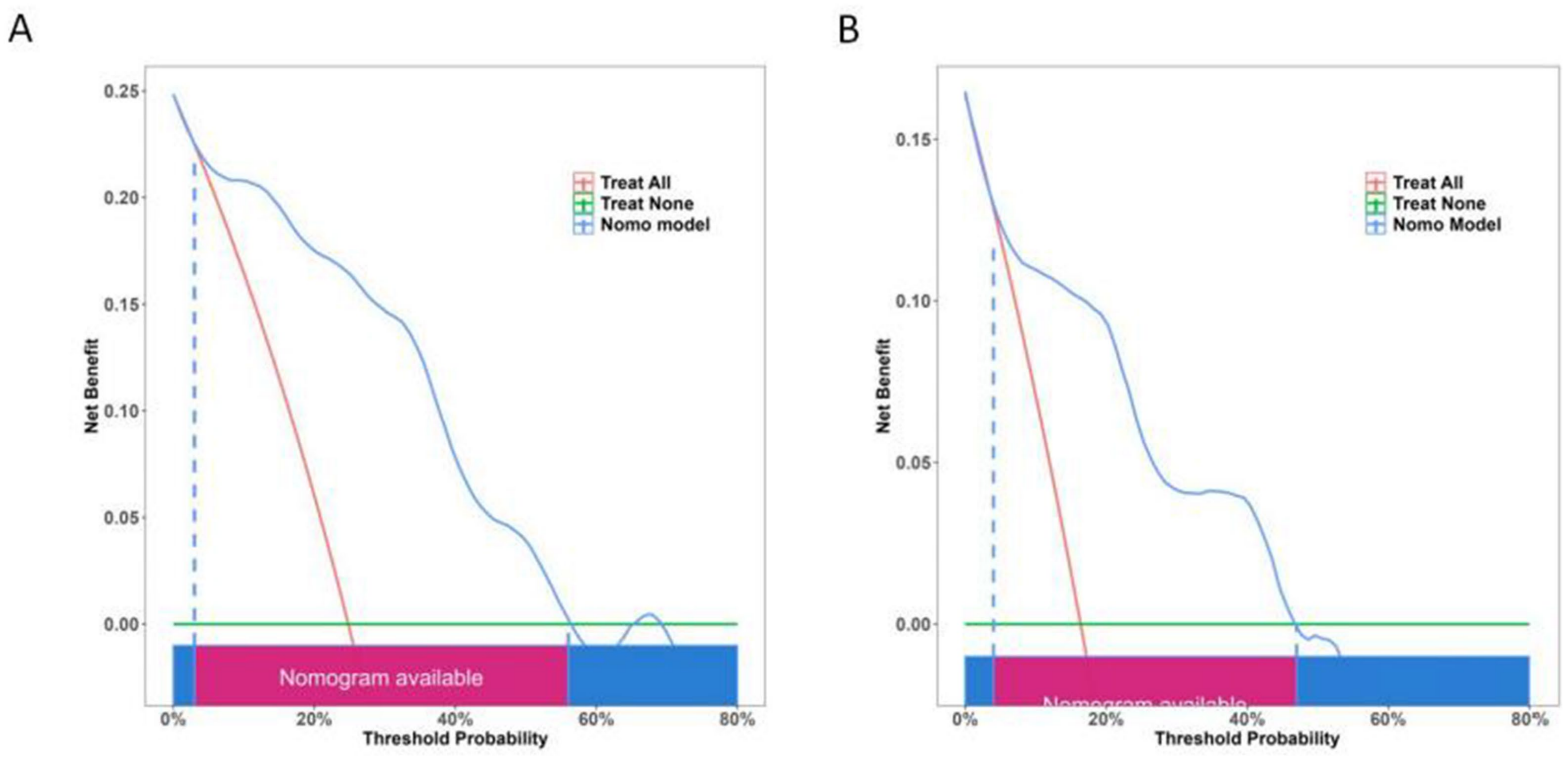
Decision curve showing the clinical net benefit of the model across different risk thresholds. **(A)** The training set showing superior clinical net benefit when the individual threshold probability ranges from 5 to 57%. **(B)** The test set showing superior clinical net benefit when the individual threshold probability falls between 7 and 47%.

DCA further highlighted the model’s advantage in predicting chronic hydrocephalus when the threshold probability ranged from 7 to 47% in the test set (Figure 12).

**Figure 13:**
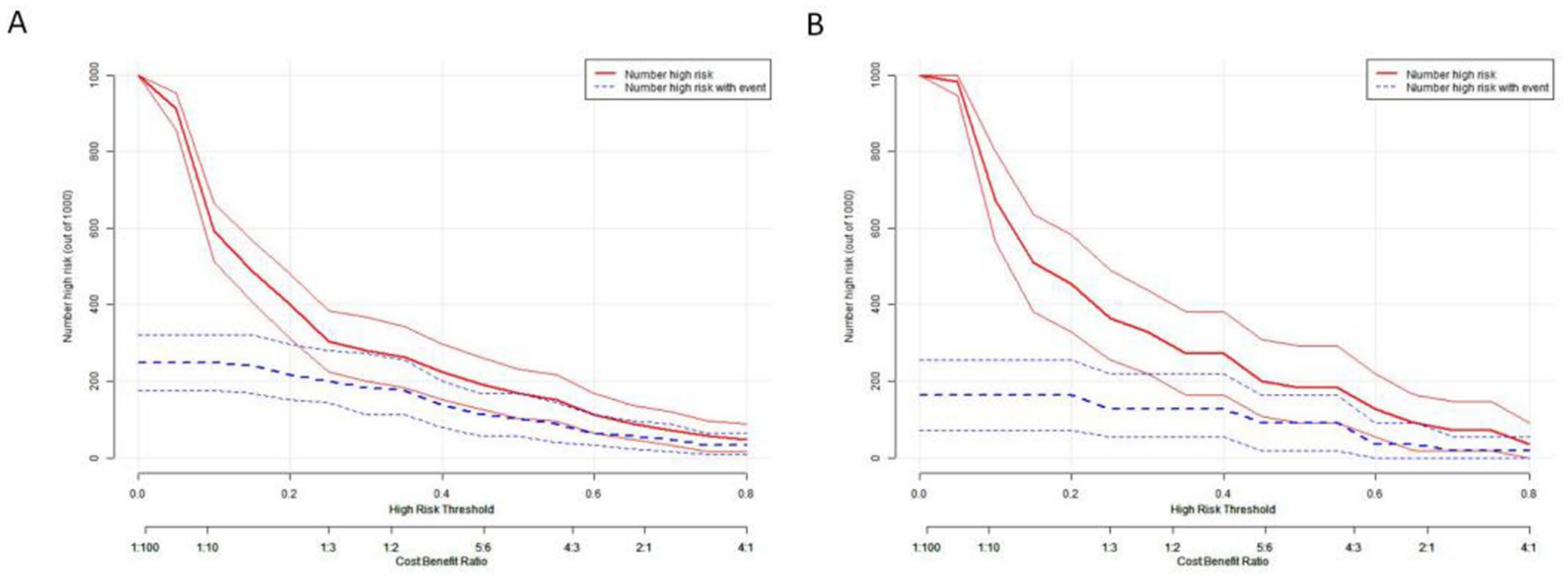
Clinical impact curve depicting the relationship between predicted high-risk individuals and actual outcomes across different thresholds. This curve aids in assessing the clinical impact of the model across various thresholds, thereby providing guidance for clinical decision-making. **(A)** The training set. **(B)** The test set.

The clinical impact curve (CIC) demonstrated the consistency of the model in predicting the number of high-risk patients for hydrocephalus compared to those actually diagnosed with the condition (Figure 13).

## 4 Discussion

This study has developed a clinical and radiological nomogram, providing a valuable tool for accurately predicting chronic hydrocephalus in individuals diagnosed with aSAH. The nomogram served as a foundation for frontline healthcare practitioners to predict the risk of chronic hydrocephalus and develop personalized treatment plans. Importantly, the model’s ROC analysis demonstrated exceptional discriminative power and calibration, with an AUC of 0.860 (95% CI: 0.758–0.962). Additionally, DCA revealed significant net clinical benefit from using this model in both the training and validation cohorts.

Multiple mechanisms are believed to contribute to the pathogenesis of chronic hydrocephalus following aSAH, including alterations in cerebrospinal fluid dynamics, obstruction of arachnoid granulations by blood metabolites, and fibrosis of the lateral ventricles (21, 29).

### 4.1 Electrolyte imbalances and osmotic effects

Patients with aSAH commonly experience electrolyte disturbances during hospitalization. This study identified a significant association between hypernatremia and the development of chronic hydrocephalus. Hypernatremia frequently manifests early post-SAH (30, 31), and may result from hypothalamic dysfunction, which impairs arginine vasopressin secretion. This condition is characterized by elevated extracellular sodium levels, leading to hyperosmolarity, with approximately 90% of the body’s sodium ions localized in the extracellular space (32). In a hyperosmolar state, water molecules within the cells move to the extracellular space to maintain osmotic balance, which reduces cell volume and leads to the contraction of brain parenchyma. To compensate for the resulting changes in intracranial pressure, cerebrospinal fluid volume increases (33). aSAH also induces hypokalemia via catecholamine-mediated β2-adrenergic stimulation of Na^+^/K^+^-ATPase, resulting in intracellular potassium shift (34–36).

Sodium ions are the dominant component of plasma osmolarity. Their acute elevation often leads to radiographically apparent ventricular enlargement, which is typically associated with low or normal intracranial pressure and can be reversed upon correction of the sodium imbalance. This condition is easily misdiagnosed as shunt failure in clinical settings, warranting particular caution in shunt-dependent patients (37, 38). Mechanistically, blood hyperosmolarity drives water movement out of brain cells, reducing brain volume and tissue tension while increasing intracranial compliance. The resulting compensatory space is occupied by CSF, leading to osmotic ventricular enlargement—a process consistent with the rapid reduction of intracranial pressure observed with hypertonic saline administration (39, 40). At the microscopic level, the choroid plexus establishes transepithelial electrochemical and osmotic gradients through Na^+^/K^+^-ATPase, carbonic anhydrase, and the Na^+^-K^+^-2Cl^−^ cotransporter, facilitating water influx into the ventricles via channels such as AQP1 and generating nearly iso-osmotic CSF. Animal and molecular studies indicate that AQP1 deficiency reduces CSF production and intracranial pressure, while NKCC1-mediated ion-water cotransport significantly contributes to CSF formation (41–43). Therefore, hypernatremia-associated ventricular enlargement should be managed through timely correction of electrolyte and osmotic imbalances.

These findings were consistent with our study, suggesting that hypernatremia in aSAH may contribute to the development of chronic hydrocephalus. Therefore, electrolyte levels should be closely monitored during hospitalization, and any imbalances should be promptly corrected.

### 4.2 CSF lactate as a predictive biomarker

Lactic acid elevation in CSF can be caused by various central nervous system disorders, including acute intracranial infections, stroke, SAH, seizures, and mitochondrial diseases (44–46). In aSAH, obstruction of fourth ventricular CSF outflow, subarachnoid erythrocytosis, and hemolysis elevate CSF lactate levels. Consistent with our findings, previous studies have shown that increased CSF lactate levels in the subarachnoid space predict the development of hydrocephalus (45). In addition, lactic acid may be produced within brain tissue and diffuse into the CSF space, explaining the difference in lactate levels between blood and CSF. High levels of extracellular lactate are primarily related to hyperglycolysis in the brain rather than hypoxia (46). Some studies suggest that the increased lactate concentration in CSF is related to protein and white blood cell count, indicating that local inflammation and blood–brain barrier dysfunction may play a role in increased CSF lactate levels (47, 48). Other research has shown that preoperative CSF glucose levels <2.8 mmol/L and CSF lactate levels >2.8 mmol/L are risk factors for infection after neurosurgery (47, 48). Furthermore, intracranial infection can affect the function of glucose transporters, decreasing the transport of glucose from blood to CSF. Under normal circumstances, the lactate content in the CSF is low, as it is mainly a product of anaerobic glucose fermentation within the CSF. CSF lactate levels are not easily influenced by peripheral blood lactate levels, making it a reliable indicator of metabolic state in the brain. High glucose levels in CSF can lead to fluid transfer from brain parenchymal cells to the extracellular space, resulting in ventricular enlargement (21, 49). However, this study did not find a significant correlation between glucose levels and chronic hydrocephalus. Therefore, CSF lactate is a more reliable biomarker for predicting chronic hydrocephalus post-aSAH.

### 4.3 Neuroimaging predictors

#### 4.3.1 Conventional metrics

Although admission CT is effective in diagnosing SAH, predicting the development of chronic hydrocephalus remains a challenge. The initial CT scan at admission plays a crucial role in diagnosing SAH, but clinicians and radiologists lack reliable tools for predicting chronic hydrocephalus. Several studies have shown that the Evans index, callosal angle (CA), temporal horn size, third ventricle width, and third ventricle diameter can provide some insight into the formation of hydrocephalus. Consistent with our findings, the CA is a key determinant in differentiating idiopathic normal pressure hydrocephalus (iNPH) from Alzheimer’s disease (AD) and other secondary causes of hydrocephalus (50, 51). A CA of less than 90° typically indicates hydrocephalus, while a CA greater than 90° suggests brain atrophy (52). CT scans can also reveal changes in the periventricular white matter regions. Although these changes may not be significant on the initial non-contrast CT scan, some patients with hydrocephalus may exhibit decreased CT attenuation in the white matter, suggesting a pressure effect from hydrocephalus (53).

#### 4.3.2 Advanced imaging and radiomics

In the study by Lee et al., diffusion tensor imaging revealed increased fractional anisotropy and mean diffusivity values in the white matter near the anterior horns of the lateral ventricles and the posterior limbs of the internal capsules in patients with iNPH. This was attributed to the potential compression of neuronal connections or vascular structures in the periventricular tissues caused by ventricular dilation, disrupting white matter tracts (54). GyeongMo Sohn’s study found that the ratio of CT attenuation between the hypothalamus and the periventricular white matter was significantly higher in patients with iNPH (55). This is due to axonal damage and increased water content in the periventricular regions, leading to decreased CT attenuation in the white matter surrounding the lateral ventricles (56). Based on these findings, we explored early changes in brain white matter associated with the formation of chronic hydrocephalus using radiomics approaches. A radscore, calculated from seven radiomic features, was significant in univariate logistic analysis but did not show significant differences in LASSO analysis. Therefore, the radscore was not included in the construction of our model. This could be due to an insufficient sample size or collinearity among variables. Nevertheless, we believe that radiomics holds great significance for predicting the formation of hydrocephalus in the future.

### 4.4 Hemorrhage severity and clinical implications

Studies by Hu et al. and Gluski et al. have shown that the total volume of intracerebral hemorrhage is closely related to acute hydrocephalus (57, 58). A study by Mijderwijk et al. demonstrated that a high Fisher grade at admission was associated with chronic hydrocephalus (15). This association occurred because bleeding triggered an inflammatory response that stimulated the arachnoid membrane, leading to substantial protein deposition and abnormal cerebrospinal fluid circulation (17). In addition, a high Fisher grade is often linked to a longer hospital stay (59). Our findings were consistent with these studies, showing that larger hemorrhage volumes could lead to slower hematoma absorption, increasing the risk of developing chronic hydrocephalus.

Severe SAH, characterized by extensive and irregular morphology, makes the traditional Fisher grade susceptible to inter-observer variability (60). It is difficult for clinicians to quantitatively assess the irregular shape and extent of the hemorrhage. In contrast, the 3D-Unet automatic segmentation model we developed provides precise quantification of the hemorrhage, providing clinicians with a clearer understanding of the patient’s condition rather than relying on a subjective grade assessment. This approach helps clinicians make better-informed decisions regarding the need for drainage surgery. Our 3D-Unet segmentation model provides objective quantification of hemorrhage volume, facilitating accurate risk stratification for drainage surgery.

## 5 Limitations of the study

However, this study had a relatively small sample size, which may have limited the diversity of the target group. Furthermore, it was a single-center study without external validation. Thus, the model’s generalizability remains uncertain. The lack of external validation means the model’s applicability to broader populations has not yet been confirmed. In the future, we plan to conduct a multicenter, prospective cohort study that includes patients from diverse regions and under varying clinical workflows to validate the model’s general applicability. In addition, the radscore we explored did not yield significant results when screening all indicators, which was somewhat contrary to our initial expectations. Besides the sample size, possible interactions among various indicators could have influenced these outcomes. This study exclusively performed radiomics analysis relying solely on computed tomography (CT) images and did not integrate other imaging modalities like magnetic resonance imaging (MRI). CT, being a frequently utilized imaging examination for patients with aneurysmal subarachnoid hemorrhage (aSAH), nonetheless has certain limitations in soft tissue resolution. This drawback may potentially compromise the accuracy of feature extraction.

We developed this machine learning model to predict the optimal treatment strategy, aiming to prevent complications associated with unnecessary treatments or delayed surgical interventions, thereby reducing both the duration and cost of hospitalization and rehabilitation. In future research, it would be advisable to attempt the integration of multimodal imaging data. By incorporating data from various imaging modalities, the accuracy of the predictive model can be further enhanced, thereby enabling more precise predictions and better - informed clinical decision - making.

## 6 Conclusion

Chronic hydrocephalus and its associated complications impose a significant burden on patients with aSAH. Due to the insidious onset of the condition, conventional clinical and imaging methods often fail to detect chronic hydrocephalus in its early stages. This study analyzed the clinical and imaging risk factors for chronic hydrocephalus and, through ML model analysis, evaluated the effectiveness of various algorithms. A nomogram incorporating both clinical and imaging indicators was constructed, offering an accurate and reliable tool for predicting the development of chronic hydrocephalus in patients with aSAH. This nomogram generates personalized disease risk probabilities for each patient through quantitative calculations. In the prediction of chronic hydrocephalus, by incorporating the patients’ risk factors, it is capable of precisely assessing their risk of developing the disease. This approach breaks through the limitations of previous judgments based on experience or a single indicator, providing the clinical field with more detailed and accurate information.

The quantitative risk information provided by the nomogram may prompt physicians to alter their original treatment strategies. For patients at a high risk of chronic hydrocephalus, cerebrospinal fluid drainage can be carried out at an early stage, or adjuvant treatment with nerve-protecting medications can be administered.

## Data Availability

The raw data supporting the conclusions of this article will be made available by the authors, without undue reservation.

## Glossary

ML: machine learning
aSAH: aneurysmal subarachnoid hemorrhage
ROC: receiver operating characteristic
AUC: area under the curve
P-R: plotting precision-recall curves
DCA: decision curve analysis
CIC: clinical impact curves
DSC: Dice similarity coefficient
IoU: intersection over union
HD: Hausdorff distance
ASSD: average symmetric surface distance
LR: logistic regression
CNNs: convolutional neural networks
GCS score: Glasgow Coma Scale
WFNS grade: World Federation of Neurosurgical Societies
V-P shunt: ventriculoperitoneal
CSF: cerebrospinal fluid
C-CRP: reactive protein
iNPH: idiopathic normal pressure hydrocephalus
XGB: eXtreme Gradient Boosting
LGBM: Light Gradient Boosting Machine
RF: Random Forest
SVM: Support Vector Machine
DT: Decision Tree
KNN: K-Nearest Neighbors
