# Supplementary material for: Machine learning in developing a predictive model for chronic hydrocephalus following aneurysmal subarachnoid hemorrhage
Source: Front Neurol. 2026 Jan 12;16:1651694. doi: 10.3389/fneur.2025.1651694 (PMC12832845; doi:10.3389/fneur.2025.1651694)
Supplement: Supplementary file 1 [file Presentation_1.pdf]

Supplement material:

Part1：Radiomics

Radiomics refers to the process of extracting a vast array of subtle features from medical imaging data that are imperceptible to the naked eye, leveraging computational techniques[1]. These features undergo quantification and screening, during which the most valuable imaging characteristics are retained for the purpose of constructing machine learning models aimed at diagnosis or outcome prediction[2].

Region of interest（ROI） drawing: A total of 180 CT scan images from patients meeting the inclusion criteria were collected. All images were acquired on the seventh day of hospital admission, following a standardized cranial CT scanning protocol, which included a tube voltage of 120 kV, a tube current of 300 mA, an image resolution of 512 × 512 pixels, and a slice thickness of 5 mm. For this study, the periventricular white matter within a 1-cm diameter surrounding the anterior horns of both lateral ventricles was designated as the region of interest (ROI). The CT plain scan images of the enrolled patients were imported into 3D Slicer software (version 5.2.2) in DICOM format[3]. The ROI delineation was performed by experienced resident physicians and subsequently verified by attending radiologists with over two decades of expertise in the diagnosis of cerebral diseases.

The specific criteria for ROI drawing were as follows:

1. **Selection of Slices**: The layer corresponding to the maximum distance between the bilateral frontal horns was selected for delineation.
2. **Adherence to Ventricular Wall**: The delineated area was closely adherent to the wall of the lateral ventricles.
3. **Exclusion of Cerebrospinal Fluid and Blood**: Care was taken to avoid inclusion of cerebrospinal fluid and blood within the ROI.

Feature Extraction:

We utilized the open-source software PyRadiomics 2.2.0 (https://github.com/Radiomics/pyradiomics) to extract radiomic features from the white matter surrounding the anterior horns of the lateral ventricles. After excluding morphological features, a total of 1395 radiomics features were analyzed within each ROI, there are a total of 2,790 radiomics features extracted from both sides. These features include first-order features, texture features, as well as those obtained after wavelet transformation, exponential and square root mathematical transformations.

encompassing 540 first-order features, 720 Gray Level Co-occurrence Matrix (GLCM) features, 480 Gray Level Run Length Matrix (GLRLM) features, 480 Gray Level Size Zone Matrix (GLSZM) features, 420 Gray Level Dependence Matrix (GLDM) features, and 150 Neighbourhood gray-tone difference matrix (NGTDM) features. Number and ratio of radiomics features .（**Supplementary Figure 1**）

Feature Selection:

The extracted features from both the left and right sides were collectively subjected to the Mann-Whitney U test to compare the feature differences between the two patient groups. Features with no significant differences ($P \geq 0.05$) were eliminated. Subsequently, Least Absolute Shrinkage and Selection Operator (LASSO) regression was employed to determine the final set of radiomic features to be included. A 10-fold cross-validation was utilized to identify the optimal regularization parameter (λ), yielding an intercept (α) and corresponding coefficients (β) for each selected feature, which were used in subsequent analyses（**Supplementary Figure 2**）.

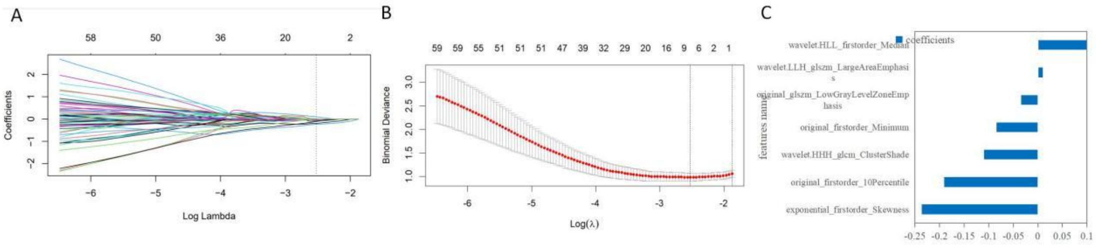

Supplementary Figure 2：Radiomic feature selection through LASSO regression. **A:** LASSO feature coefficient path plot showing the variation of feature coefficients as a function of the regularization parameter. **B:** LASSO cross-validation curve illustrating the mean squared error (or another appropriate metric) as a function of the regularization parameter, used to select the optimal value. **C**:Final retained radiomic features and their corresponding coefficients.

The final features and their corresponding coefficients included in the subsequent study are in **Supplementary Figure 3** and **Supplementary Table 1.**

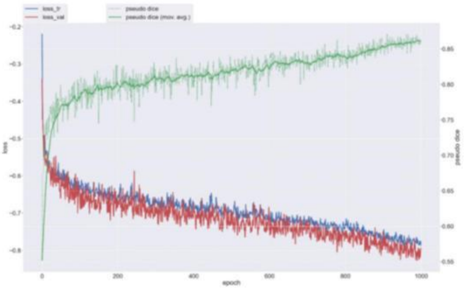

Supplementary Figure 3: Training process for the automatic segmentation model, with curves showing the changes in average loss and Dice coefficient over the course of the training epochs.

The Rad-score calculation formula is as follows[4]:

$$Rad - score = \alpha + \sum_{1}^{i} \beta_i X_i$$

α is the intercept, β is the coefficient corresponding to the radiomics features, and X is the value of the radiomics features.

Part2：3D-Unet

Data Preprocessing: The image resolution was initially set at 512 × 512 pixels, with a slice thickness of 5 mm. To ensure uniformity in spatial spacing, all data were resampled to a uniform spatial thickness of 0.5 × 0.5 × 5 mm. Subsequently, the brain was extracted from the raw CT images, with the skull considered an irrelevant background and subsequently removed. Following brain extraction, slices with less than 10% of effective brain tissue area at the sequence extremities, where hemorrhage is infrequent, were discarded. A resident physician with 3 years of experience in image diagnosis manually annotated the full extent of hemorrhage lesions within the scan range using ITK-SNAP software. Subsequently, a chief physician with over 10 years of experience in image diagnosis reviewed and corrected these annotations for

confirmation. The dataset was then partitioned into training, internal validation, and internal testing sets in a 7:1:2 ratio. Horizontal flipping of the training dataset was applied to enhance data diversity.

Framework Construction: The 3D-UNet (**Figure 3**), a deep learning-based segmentation approach, was employed to construct an adaptive aSAH automatic segmentation model. This novel network structure dynamically adjusts based on dataset characteristics, encompassing preprocessing, network architecture, training, and post-processing phases. Utilizing the 3D-UNet, 144 manually segmented cases were incorporated into the training set, with segmentation regions encompassing the entire lesion layer. The model's efficacy was subsequently evaluated using 36 test set cases.

Upon completing the data preprocessing steps, the 3D-UNet network dynamically optimizes and tunes its critical hyperparameters during training, catering to the preprocessed data's unique characteristics. The model's core architecture comprises two primary components: the encoder and decoder, which collaboratively process and reconstruct the data. In designing the architecture, we meticulously selected a feature channel sequence of [32, 64, 128, 256, 320] to construct the encoding and decoding hierarchical structure. This pathway enables the initial 512x512 pixel input images to be progressively downsampled to 32x32, followed by an upsampling process that gradually restores them to their original dimensions, thereby extracting and refining image information layer by layer.

Our established 3D-UNet automatic segmentation model features a network architecture where each layer of the encoder and decoder is composed of two convolutional blocks (Conv Blocks). The activation function employed is the Rectified Linear Unit (ReLU), and the convolutional kernels are set to 3×3×3. Through downsampling, 320 high-level features are extracted, which are then laterally reversed from the downsampling path to the upsampling path. Subsequently, the corresponding upsampling restores the dimensions, ultimately outputting the automatically segmented image. The model's initial learning rate is set to 0.01, and it undergoes 1000 epochs of training.

During the training phase, to enhance the model's generalization capability, we adopted a diverse set of data augmentation strategies. These strategies encompass data flipping, multi-angle rotation (randomly selected angles ranging from -30° to 30° along the X, Y, and Z axes), scaling adjustments (within a ratio of 0.7 to 1.4), and γ correction (with an adjustment range of 0.7 to 1.5). Additionally, to further boost data diversity, we introduced transformations such as the addition of Gaussian noise, Gaussian blurring, and mirroring. Notably, all these data augmentation techniques were strictly applied within the training dataset to ensure the independence of the test set and fairness in evaluation.

In segmentation tasks, the Dice Loss or Cross-Entropy Loss functions are commonly employed to reflect the model's training process and performance. The Dice Loss, derived from the Dice coefficient, is inversely correlated with the model's Dice Similarity Coefficient (DSC). The Cross-Entropy Loss, on the other hand, quantifies the discrepancy between the model's predicted distribution and the true distribution.

In the Dice Loss function, Nrepresents the total number of voxels, p_i denotes the probability predicted by the automatic segmentation model that the i-th voxel belongs to the target class, and g_i is the probability that the i voxel in the manually segmented set belongs to the target class.

For the Cross-Entropy Loss function, M signifies the total number of classes, which in this study equals 2. y_c represents the true probability distribution for the c class, while p_c denotes the predicted probability distribution for the c class in the predicted samples. y_c takes a value of 1 if the c class matches the sample's class, and 0 otherwise.

$$L_{dice} = -\frac{2\sum_{i=1}^{N} p_i g_i}{\sum_{i=1}^{N} p_i^2 + \sum_{i=1}^{N} g_i^2}$$

$$L_{ce} = -\sum_{c=1}^{M} y_c \log(p_c)$$

In this study, we employed a hybrid loss function L, which combines Dice Loss (L_{dice}) and Cross-Entropy Loss (L_{ce}). The weights for L_{dice} and L_{ce}, denoted as ω_{dice} and ω_{ce}, respectively, were both set to 0.5.

The model was optimized using the Stochastic Gradient Descent (SGD) optimizer with a momentum coefficient of 0.99, aimed at accelerating convergence in relevant directions and suppressing oscillations. The initial learning rate ($initial\_lr$) was set to 0.01, and the learning rate (lr) was adjusted during training epochs. To prevent overfitting, a weight decay ($weight\_decay$) of 0.00003 was applied.

Upon completion of training, 36 images were used as the test set to quantitatively evaluate the segmentation performance of each model using Dice Similarity Coefficient (DSC), Intersection over Union (IoU), Hausdorff Distance (HD), and Average Symmetric Surface Distance (ASSD)[5, 6]. The results of the 36 test cases are provided in **Supplement Table 1.**

Evaluation parameters for automatic segmentation models

1.**Dice Similarity Coefficient (DSC):** As a quantitative metric, DSC is utilized to evaluate the degree of similarity between two sets, X and Y. In this study, DSC is

specifically applied to compare the level of agreement between manually segmented and automatically segmented regions. Its value ranges from 0 to 1, providing an intuitive and easily interpretable scale. A DSC value closer to 1 indicates a higher similarity in segmentation results, signifying optimal segmentation performance. Conversely, a DSC value approaching 0 implies the most significant differences between segmentation outcomes, indicating poor segmentation. Therefore, DSC serves as a crucial parameter for assessing the quality of segmentation performance.

$$DSC = \frac{2|X \cap Y|}{X \cup Y} = \frac{2TP}{2TP + FN + FP},$$

2.**Intersection over Union (IoU):** A key indicator for evaluating the spatial overlap between a predicted region X and a ground truth region Y, IoU in this study is specifically defined as the ratio of the intersection area between the automatically segmented region and the manually annotated region to their union area. It directly reflects the proportion of voxel overlap between the prediction and the true label. The value of IoU ranges from [0,1], with a value closer to 1 indicating a higher degree of overlap between prediction and ground truth, thus closer to ideal segmentation performance. Conversely, an IoU value tending towards 0 signifies significant differences between the predicted and true regions, indicating poor segmentation performance. Therefore, IoU is an important criterion for measuring segmentation precision and accuracy.

$$Io\,U\,(X,Y) = \frac{X \cap Y}{X + Y - X \cap Y} = \frac{TP}{TP + FN + FP}$$

3. **Average Symmetric Surface Distance (ASSD):** An index that quantifies the overall trend of the closest distances between the surfaces of two segmentation results, with the minimization of this average value indicating better segmentation consistency. Specifically, let X and Y be the two regions to be compared. For any point x in X and any point y in Y, d(y,x) is defined as the spatial distance between these two points. By calculating the minimum distance between each pair of points and taking the average of the minimum distance set, the overall average of the shortest distances between the entire regions is obtained. len(X) and len(Y) represent the lengths of regions X and Y, respectively. A smaller ASSD value implies a higher degree of matching between the surface morphologies of the two segmentation results, reflecting better segmentation quality.

$$ASSD = \frac{\sum_{x \in X} min_{y \in Y} d(x,y) + \sum_{y \in Y} min_{x \in X} d(y,x)}{len(X) + len(Y)}$$

4. **Hausdorff Distance (HD):** A measure that describes the similarity between two

sets of points, effectively assessing the differences between model predictions and ground truth, particularly in shape matching and boundary accuracy evaluation. It is a form of defining the distance between two point sets: Given two sets A={a1,…,ap} and B={b1,…,bq}, the Hausdorff distance between these two point sets is defined as:

$$H(A,B) = \max\big(h(A,B), h(B,A)\big)$$

$$\cdot h(A,B) = \max_{a\in A}\Big\{\min_{b\in B} \| a - b \|\Big\}$$

$$\cdot h(B,A) = \max_{b\in B}\Big\{\min_{a\in A} \| b - a \|\Big\}$$

$\|\cdot\|$ denotes the distance between two points.

Part3： All models

Supplementary Table1: The results of Brier scores for all models.

model	brier_score	group
XGboost	0.0824 (0.0637-0.1133)	train
XGboost	0.1402 (0.0916-0.2096)	test
SVM	0.1556 (0.1232-0.1951)	train
SVM	0.1292 (0.0917-0.1772)	test
RF	0.0200 (0.0156-0.0263)	train
RF	0.1479 (0.1030-0.2095)	test
NBM	0.1415 (0.0988-0.1954)	train
NBM	0.2074 (0.1323-0.3180)	test
LR	0.1313 (0.0991-0.1722)	train
LR	0.1201 (0.0807-0.1814)	test
LGBM	0.1865 (0.1462-0.2268)	train
LGBM	0.1440 (0.0890-0.1898)	test
KNN	0.0270 (0.0196-0.0356)	train
KNN	0.1838 (0.1151-0.2719)	test
DT	0.1125 (0.0823-0.1558)	train
DT	0.1497 (0.0999-0.2275)	test

Supplementary Table2: The results of various evaluation indicators for all models.

model	group	Sensitivity	Specificity	Pos Pred Value	Neg Pred Value	Precision	Recall	F1	Prevalence	Balanced Accuracy
XGboost	train	0.903	0.894	0.737	0.966	0.737	0.903	0.812	0.248	0.898
XGboost	test	0.556	0.739	0.294	0.895	0.294	0.556	0.385	0.164	0.647
SVM	train	0.871	0.766	0.551	0.947	0.551	0.871	0.675	0.248	0.818
SVM	test	1	0.609	0.333	1	0.333	1	0.5	0.164	0.804
RF	train	1	1	1	1	1	1	1	0.248	1
RF	test	0.444	0.783	0.286	0.878	0.286	0.444	0.348	0.164	0.614
NBM	train	0.903	0.723	0.519	0.958	0.519	0.903	0.659	0.248	0.813
NBM	test	0.778	0.609	0.28	0.933	0.28	0.778	0.412	0.164	0.693
LR	train	0.871	0.819	0.614	0.951	0.614	0.871	0.72	0.248	0.845
LR	test	1	0.696	0.391	1	0.391	1	0.562	0.164	0.848
LGBM	train	1	0	0.248		0.248	1	0.397	0.248	0.5
LGBM	test	1	0	0.164		0.164	1	0.281	0.164	0.5
KNN	train	1	1	1	1	1	1	1	0.248	1
KNN	test	0.222	0.783	0.167	0.837	0.167	0.222	0.19	0.164	0.502
DT	train	0.676	0.912	0.742	0.883	0.742	0.676	0.708	0.272	0.794
DT	test	0.357	0.902	0.556	0.804	0.556	0.357	0.435	0.255	0.63

[1] LAMBIN P, RIOS-VELAZQUEZ E FAU - LEIJENAAR R, LEIJENAAR R FAU - CARVALHO S, et al. Radiomics: extracting more information from medical images using advanced feature analysis [J]. (1879-0852 (Electronic)).

[2] GUIOT J, VAIDYANATHAN A, DEPREZ L, et al. A review in radiomics: Making personalized medicine a reality via routine imaging [J]. (1098-1128 (Electronic)).

[3] FEDOROV A, BEICHEL R FAU - KALPATHY-CRAMER J, KALPATHY-CRAMER J FAU - FINET J, et al. 3D Slicer as an image computing platform for the Quantitative Imaging Network [J]. (1873-5894 (Electronic)).

[4] HUANG Y Q, LIANG C H, HE L, et al. Development and Validation of a Radiomics Nomogram for Preoperative Prediction of Lymph Node Metastasis in Colorectal Cancer [J]. (1527-7755 (Electronic)).

[5] TAHA A A, HANBURY A. Metrics for evaluating 3D medical image segmentation: analysis, selection, and tool [J]. (1471-2342 (Electronic)).

[6] VINAYAHALINGAM S, KEMPERS S, SCHOEP J, et al. Intra-oral scan segmentation using deep learning [J]. (1472-6831 (Electronic)).
